# Inflammasome Meets Centrosome: Understanding the Emerging Role of Centrosome in Controlling Inflammasome Activation

**DOI:** 10.3389/fimmu.2022.826106

**Published:** 2022-02-24

**Authors:** Dandan Wu, Zhenzhen Zhang, Xiaoli Jiang, Yaning Du, Shuangyan Zhang, Xiao-Dong Yang

**Affiliations:** ^1^ Department of Immunology and Microbiology, Shanghai Institute of Immunology, Shanghai Jiao Tong University School of Medicine, Shanghai, China; ^2^ Department of Biochemistry and Molecular Cell Biology, Shanghai Jiao Tong University School of Medicine, Shanghai, China

**Keywords:** inflammasome, centrosome, NLRP3, pyrin, assembly, activation

## Abstract

Inflammasomes are multi-protein platforms that are assembled in response to microbial and danger signals to activate proinflammatory caspase-1 for production of active form of IL-1β and induction of pyroptotic cell death. Where and how an inflammasome is assembled in cells has remained controversial. While the endoplasmic reticulum, mitochondria and Golgi apparatus have been reported to be associated with inflammasome assembly, none of these sites seems to match the morphology, number and size of activated inflammasomes that are microscopically observable as one single perinuclear micrometer-sized punctum in each cell. Recently, emerging evidence shows that NLRP3 and pyrin inflammasomes are assembled, activated and locally regulated at the centrosome, the major microtubule organizing center in mammalian cells, elegantly accounting for the singularity, size and perinuclear location of activated inflammasomes. These new exciting findings reveal the previously unappreciated importance of the centrosome in controlling inflammasome assembly and activation as well as inflammasome-related diseases.

## Introduction

Canonical inflammasomes are multi-protein complexes that typically assemble from a sensor protein, the adaptor protein ASC and the effector protein procaspase-1 in host cells (1, 2). When responding to both exogenous pathogen-associated molecular patterns (PAMPs) and endogenous damage-associated molecular patterns (DAMPs), inflammasomes assemble to activate caspase-1 for the cleavage of interleukin-1β (IL-1β) precursor and the pore-forming protein gasdermin D (3), resulting in IL-1β maturation, pyroptotic cell death and subsequently potent inflammatory response (4).

Based on the type of sensor proteins, canonical inflammasomes can be grouped into three major families: the NLRP family, the NLRC family, and non-NLR family. When being assembled, sensor proteins of the NLRP family, including NLRP3, NLRP1, NLRP6 and NLRP12 inflammasomes, and the non-NLR inflammasomes, such as the bacterial toxin-sensing pyrin inflammasome and the DNA-sensing AIM2 inflammasome, can bind to adaptor protein ASC through PYD domain- or CARD domain-mediated homotypic interaction. ASC in turn recruits caspase-1 *via* CARD domain-mediated homotypic interaction. In contrast, the sensor protein of NLRC4 inflammasome of the NLRC family has a CARD domain by itself to directly engage caspase-1 but still requires ASC for optimal activation. Similarly, NLRP1 also has a CARD domain that can engage caspase-1 to bypass the requirement for ASC to activate NLRP1 inflammasome (5–7). Activation of all types of inflammasomes needs to be controlled properly; dysregulated activation of them often leads to excessive inflammation that underscores autoinflammatory diseases, cancer, neurodegeneration, and cardiometabolic disorders (8).

Regardless the types of inflammasomes and the stimuli that activate them, a well-defined remarkable hallmark of inflammasome activation is the formation of one single micrometer-sized perinuclear punctum in each cell (9, 10), observable by microscopy as a so-called ASC speck. Molecularly, this is a sensor protein-driven rapid clustering of ASC protein into a macromolecular aggregate. While extensively studied as an indication of activation of canonical inflammasomes soon after the inflammasome was initially discovered in 2002 (11), it remains a matter of debate about how the speck is formed, why only one speck is formed within a cell, and whether it is associated with any cellular structure.

## Association Of NLRP3 Inflammasome With ER, Mitochondria and Golgi Apparatus

Among inflammasomes, the NLRP3 inflammasome is unique in that it can be activated by physically and structurally diverse stimuli. Its subcellular localization is dynamic and has been intensively studied. In unstimulated cells, the NLRP3 protein is distributed in cytoplasm with a marked association with the ER (12–14). Upon stimulation by NLRP3 activators, however, it becomes associated with mitochondria or ER-mitochondria contact sites, also known as mitochondria-associated membranes (MAM) (12, 13). In support of mitochondrial localization of NLRP3, further studies show that the antiviral mitochondrial adaptor protein MAVS (13, 15) or the mitochondrial lipid cardiolipin (16, 17) can bind and recruit NLRP3 to the mitochondria where NLRP3 and ASC may interact for inflammasome assembly and activation.

The Golgi apparatus is another subcellular location that has recently been appreciated for NLRP3 inflammasome assembly and activation. Cholesterol homeostatic regulator SCAP-SREBP2 can associate with NLRP3 protein to escort the translocation of NLRP3 from ER to the Golgi apparatus for optimal inflammasome assembly in macrophages (18). In line with this finding, another study reveals that perturbation of the ER-Golgi trafficking attenuated NLRP3 inflammasome activation (19). Furthermore, in an elegantly designed *in vitro* assay using reconstituted 293T cells to study NLRP3 activity, Chen et al. demonstrates that most NLRP3 protein resides in the cytosol, only a membrane-associated form of NLRP3 becomes active after stimulation (10). They further show that different NLRP3 stimuli promote dispersion of the trans-Golgi network (TGN) and the negatively charged phosphatidylinositol-4-phosphate (PtdIns4P) exposed on the dispersed TGN (dTGN) recruit NLRP3 to form multiple puncta which induce ASC polymerization and subsequent inflammasome activation (10). A recent structural study reveals that full-length mouse NLRP3 forms an oligomeric double-ring cage *via* a LRR-LRR interaction, which is required for the formation of dTGN vesicles, providing structural insights into NLRP3-induced TGN dispersion into vesicles (20). The stimulus-induced NLRP3 recruitment to and aggregation at dTGN can also be seen in primary ASC-deficient bone marrow-derived macrophages (BMDMs), indicating that, in response to diverse stimuli, recruitment of NLRP3 to dTGN may be an early and common cellular event that leads to NLRP3 aggregation before the final assembly and activation (10). Interestingly, it has been documented that multiple small-sized puncta, instead of a single speck, can also promote NLRP3 inflammasome activation induced by unconventional stimulators, such as H_2_O_2_ and MSU (21). These small puncta, so-called small “death complexes”, have also been observed during activation of NLRC4 and AIM2 inflammasomes (22, 23).

In addition to dTGN vesicles, endosomal vesicles also have PtdIns4P on the membrane (24) and may serve as alternative sites for NLRP3 to form aggregates prior to inflammasome activation (25). It has been reported that NLRP3 activators can disrupt endosome-TGN retrograde transport (ETRT) and lead to localization of NLRP3 to endosomal vesicles, and that chemical or genetic disruption of ETRT potentiates NLRP3 enrichment on endosomal vesicles and inflammasome activation (25–27).

It is worth noting that the ER, mitochondria, Golgi apparatus and endosomal vesicles are all membrane-bound organelles. The association of NLRP3 with these organelles indicates that NLRP3 may have an ability to bind membranes. This is confirmed by a structural study which shows that both overexpressed and endogenous NLRP3 is predominantly membrane bound and that membrane binding is critical for NLRP3 double-ring cage formation by serving as a scaffolding platform (20).

## Assembly and Activation of NLRP3 and Pyrin Inflammasomes at the Centrosome

**Figure 1:**
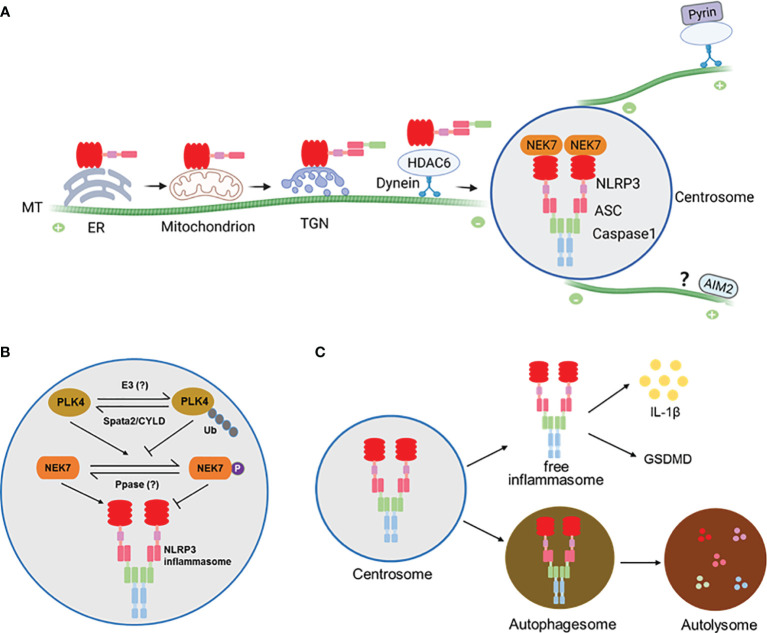
**(A)** A model for assembly and activation of inflammasomes at the centrosome. NLRP3 protein is largely associated with the ER before stimulation and becomes associated with mitochondrion or trans-Golgi network (TGN) where it is engages ASC to form complex that is trafficked to the centrosome along the microtubules (MTs) by the HDAC6-Dynein motor machinery. At the centrosome NLRP3 and ASC interact with NEK7 and Caspase1 to finish assembly and activation. The Pyrin inflammasome is similarly assembled and activated at the centrosome. It’s likely that the AIM2 inflammasome might also be activated at the centrosome via an unknown mechanism. **(B)** Regulation of NLRP3 inflammasome at the centrosome by the centrosomal deubiquitinase complex Spata2-CYLD. Spata2-CYLD mediates the deubiquitination of PLK4 to promote PLK4 phosphorylation of NEK7, which inhibits NEK7-NLRP3 interaction and thereby NLRP3 inflammasome activation. **(C)** NLRP3 inflammasome assembled at the centrosome can exit into autophagosomes that fuse with lysosomes to cause inflammasome degradation.

As described above, plenty of work has suggested the assembly and activation of NLRP3 inflammasome in the ER, MAM or Golgi (**Figure 1A**). However, none of these locations appears to match the morphology, number and size of activated inflammasomes, implying that these locations might be intermediate but not the final sites for inflammasome assembly and activation. Surprisingly, work by our group and others has recently demonstrated that, when stimulating human THP-1, mouse primary BMDM cells, or mouse immortalized BMDM (iBMDM) cells with NLRP3 activators, such as nigericin and MSU, NLRP3 inflammasome is assembled and activated at the centrosome (9, 28, 29), the major microtubule organizing center (MTOC) in mammalian cells. In these stimulated cells, not only NLRP3 and ASC, but active caspase-1 and IL-1β, at least partially, colocalizes with the centrosome (29), suggesting that NLRP3 inflammasome puncta formed at the centrosome serve as a major site for both caspase-1 activation and IL-1β maturation. Not come singly but in pairs, activation-induced pyrin inflammasome puncta also colocalize with the centrosome in iBMDMs (29). In contrast, dsDNA-induced AIM2 inflammasome puncta, formation of which also depends on ASC as NLRP3 and pyrin inflammasomes do, stay away from the centrosome in iBMDMs (29). Together, these exciting findings define the centrosome as a platform for final assembly and activation of NLRP3 and pyrin inflammasomes, which is further supported by the evidence summarized below.

The centrosome is normally positioned at the periphery of the nucleus to nucleate and anchor microtubules (MTs). It is evident that MT homeostasis and MT retrograde transport by motor proteins play a central role for activation signal-elicited trafficking of NLRP3 protein to the centrosome for inflammasome assembly (29, 30). Pretreatment of cells with inhibitors for MT, retrograde transport motor protein dynein, and the dynein adaptor, histone deacetylase HDAC6, all block the NLRP3 activator-induced generation of inflammasome puncta and inflammasome activity (29). Furthermore, similar to what can be seen for ASC deficiency, HDAC6 deficiency leads to accumulation of multiple NLRP3 speckles at the TGN (29). These findings indicate that motor protein dynein and its adaptor HDAC6-mediated retrograde transport along the MTs is required for NLRP3 trafficking to and inflammasome assembly at the centrosome. Interestingly, the MT-affinity regulating kinase 4 (MARK4), another regulator of MT-based transport, is also involved in the process of NLRP3 localization to the centrosome (9). Deficiency of MARK4 or disruption of MARK4-NLRP3 interaction impairs NLRP3 inflammasome speck formation and activation (9).

It is noteworthy that the MT inhibitor Colchicine can efficiently suppress NLRP3 inflammasome activation only when a lower concentration of NLRP3 stimulators is applied (21, 31, 32). Surprisingly, upon stimulation with higher concentrations of these stimulators, caspase-1 activation and IL-1β production cannot be effectively blocked by Colchicine while NLRP3-ASC speck formation can be abolished completely under the same condition (21). This finding indicates that when the stimulation is strong, the MT-dependent NLRP3-ASC speck formation is no longer essential for NLRP3 activation and IL-1β production which may happen in an ASC speck-independent manner. This is consistent with the observation that ASC speck formation and NLRP3 inflammasome activation are two separate events that can be uncoupled at least in some scenarios (21, 22).

In agreement with the importance of MTs in inflammasome assembly and activation, it has long been known that colchicine, as well as other MT-disrupting drugs, is capable of inhibiting IL-1β production and can be an effective medicine to treat gout, an NLRP3 inflammasome-related inflammatory disease (33, 34), and familial Mediterranean fever and hyperimmunoglobulinemia D syndrome, pyrin inflammasome-related inflammatory diseases (35).

## Regulation of NLRP3 Inflammasome Activation at the Centrosome

The assembly and activation of NLRP3 inflammasome at the centrosome is regulated locally by centrosomal proteins. The first reported centrosomal protein that modulates NLRP3 inflammasome activation is NEK7, a member of the NIMA-related kinase (NEK) family, which predominantly resides at the centrosome and regulates microtubule nucleation and spindle assembly (36, 37). In 2016, three groups independently found that NEK7 was required for NLRP3 inflammasome activation *via* specifically interacting with NLRP3 protein in the murine system (38–40). Further study showed that upon NLRP3 inflammasome activation, NEK7 colocalizes with inflammasome speck and gamma-tubulin, establishing NEK7 as a new essential component of NLRP3 inflammasome complex formed at the centrosome (29). However, in HDAC6 deficient cells NEK7 did not colocalize with the NLRP3 trapped at the TGN (29), suggesting that centrosome-localized NEK7 engages NLRP3 delivered to the centrosome for the final assembly of NLRP3 inflammasome.

Recent studies suggest a NEK7-independent mechanism for NLRP3 inflammasome activation in murine and, in particular, in human cells (41). Consistently, some LRR-depleted NLRP3 mutants that are presumably deficient for double-ring cage formation and unable to traffic to the centrosome to engage NEK7 are still able to form active inflammasomes (42, 43). The existence of both NEK7-dependent and NEK7-independent mechanisms for NLRP3 activation implies that when and how NEK7 engages NLRP3 for inflammasome activation could be complex and need to be further clarified.

Our recent work demonstrates that NEK7-NLRP3 engagement is down-regulated by a deubiquitinase complex that resides in the centrosome (28). As a well-known pivotal deubiquitinase that is involved in regulation of inflammatory responses, CYLD is primarily distributed in the cytosol. We show that CYLD’s partner protein Spata2 is enriched at the centrosome and recruits CYLD to the centrosome to mediate the deubiquitination of PLK4, a key kinase regulator of centrosome duplication. Intriguingly, deubiquitinated PLK4 binds and phosphorylates NEK7 at Ser204. Phosphorylation of NEK7 in turn attenuates NEK7 and NLRP3 interaction and thereby inhibits NLRP3 inflammasome activation at the centrosome. More importantly, deficiency of Spata2 or CYLD, inhibition of PLK4, or mutation of NEK7 phosphorylation site Ser204 to Ala each augments NLRP3 inflammasome activation. These findings identify a centrosome-localized, deubiquitinase complex-driven cascade that negatively modulates the final assembly and activation of NLRP3 inflammasome at the centrosome (**Figure 1B**).

## NLRP3 Inflammasome Exits the Centrosome After Activation

While assembled and activated at the centrosome, NLRP3 inflammasome appears not to stay there persistently. We show that in LPS/nigericin-stimulated primary BMDMs, NLRP3 inflammasome speck association with the centrosome is dynamic, peaking at about 20 min post stimulation by nigericin, then diminishing quickly, and finally exiting the centrosome into the cytosol (28). Not only that, the NLRP3 inflammasome speck can be even released to extracellular space where it is still functioning to amplify inflammatory responses (44).

In addition to exiting the centrosome as an intact and functional complex, NLRP3 and pyrin inflammasomes assembled at the centrosome are subject to autophagic degradation (29), a cellular mechanism that is employed to clear large ubiquitinated protein aggregates formed at the centrosome. In this regard, the centrosome acts as a hub to promote the fusion of inflammasome-containing autophagosomes with lysosomes to accelerate inflammasome degradation (29) (**Figure 1C**).

## Discussion

While controversial for a long time, it’s time to integrate all evidence and clarify the inconsistencies in understanding the assembly and activation of inflammasomes, such as NLRP3 and pyrin. Undoubtedly, the localization of inflammasome components is dynamic. In the case of NLRP3, not always behaving uniformly, but in most instances it appears to remain in the ER before activation, traffic to the MAM/mitochondria upon activating stimulations, then move to and accumulate at the TGN and possibly endosomal vesicles as well, and finally arrive at the centrosome to complete the inflammasome assembly and activation (**Figure 1A**).

The centrosome has been discovered as a cellular organelle for over 130 years, but there are still numerous puzzles between its structure and function. Apart from the well-defined function in organizing microtubules, other emerging roles have recently been appreciated. For instance, the centrosome has a role in immunological synapse formation between T cells and antigen presenting cells (45). Surprisingly, another study indicates that the centrosome is critical for inflammatory cytokine secretion in innate immune responses (46). The aforementioned studies reveal the importance of the centrosome in balancing the assembly, activation and attenuation of NLRP3 and pyrin inflammasomes, and add a new layer of complexity to functions of the centrosome in inflammatory responses. The physiological implication of the centrosome-associated inflammasome assembly and activation and the cross-talk between inflammasomes and the centrosome remain enigmatic and merit further investigation.

Since all known canonical inflammasomes form a single micrometer-sized speck upon activation, it is tempting to speculate that inflammasome assembly and activation at the centrosome might not be restricted to NLRP3 and pyrin, but represents a universal phenomenon applicable to many other inflammasomes. This speculation is at least partially supported by sporadic findings that LPS induces NLRP7 inflammasome colocalization with MTOC in human peripheral blood mononuclear cells (47), and that AIM2 inflammasome speck stimulated by poly (dA-dT) co-localizes with MTOC in NPC cells in which AIM2-mediated IL-1β secretion can be abrogated by a MT inhibitor but enhanced by a MT stabilizer (48, 49). In addition to NLRP3 and pyrin, whether and how other inflammasomes are assembled and/or activated at the centrosome are great questions and await further clarification.

## Author Contributions

X-DY conceptualized, planned, and wrote the manuscript. DW and ZZ wrote the manuscript. XJ, YD, and SZ reviewed the manuscript. All authors approved the submitted version of the manuscript.

## Funding

The work was supported by grants from the National Natural Science Foundation of China to XDY (31770818 and 31570770), the Shanghai Science and Technology Commission to XDY (21ZR1456300), and the National Key R&D Program of China (2021YFA1301400).

## Conflict of Interest

The authors declare that the research was conducted in the absence of any commercial or financial relationships that could be construed as a potential conflict of interest.

## Publisher’s Note

All claims expressed in this article are solely those of the authors and do not necessarily represent those of their affiliated organizations, or those of the publisher, the editors and the reviewers. Any product that may be evaluated in this article, or claim that may be made by its manufacturer, is not guaranteed or endorsed by the publisher.
